# Comparison of Memory Impairment and Oxidative Stress Following Single or Repeated Doses Administration of Scopolamine in Rat Hippocampus

**DOI:** 10.29252/NIRP.BCN.9.1.5

**Published:** 2018

**Authors:** Milad Rahimzadegan, Maliheh Soodi

**Affiliations:** 1. Department of Toxicology, School of Medical Sciences, Tarbiat Modares University, Tehran, Iran.

**Keywords:** Scopolamine, Oxidative stress, Acetylcholinesterase, Memory

## Abstract

**Introduction:**

Scopolamine, a muscarinic cholinergic receptor antagonist, is widely used to induce memory impairment in experimental animals. The present study aims to compare memory impairment and oxidative stress following single and repeated doses administration of scopolamine.

**Methods:**

A group of rats received a single shot of scopolamine in different doses (0.5, 1, or 3 mg/kg, IP) 24 hours after the passive avoidance training. Then the memory retrieval test was performed 30 minutes and 7 days after the injection. In the other experiment, rats received similar doses of scopolamine for 7 consecutive days, 24 hours after the training session. Then the memory retrieval test was performed 30 minutes and 7 days after the last injection. Acetylcholinesterase (AChE) activity and lipid peroxidation were measured in their hippocampus tissue, too.

**Results:**

Scopolamine administered in repeated doses caused more impairment in memory function compared to single dose injection based on the evaluation 30 minutes after injection. Moreover, the memory impairment persisted for 7 days only in repeated doses treated groups. Increase in acetylcholinesterase activity and lipid peroxidation in both groups was observed 30 minutes after scopolamine administration. These abnormal increases persisted for 7 days only in repeated doses treated groups. Increased AChE activity and lipid peroxidation was well correlated with behavioral deficit. Also AChE activity was well associated with lipid peroxidation.

**Conclusion:**

The results of present study showed that repeated administration of scopolamine induced results in memory impairment. This effect can be due to long-lasting oxidative stress which may damage the hippocampus tissue.

## 1. Introduction

Scopolamine, a tropane alkaloid isolated from plant sources, is a nonselective muscarinic antagonist, which induces memory impairment (Klinkenberg & Blokland, 2010). Central cholinergic system plays a crucial role in learning and memory functions for humans and animals. According to the cholinergic hypothesis, elderly memory dysfunction is due to loss of central cholinergic neurons and decreased levels of brain acetylcholine (Perry, 1986). Scopolamine-induced amnesia, most likely caused by a blockade of cholinergic signaling, is used as a pharmacological model of Alzheimer Disease (AD) (Ebert & Kirch, 1998). Various behavioral studies have been carried out to investigate the effect of scopolamine on learning and memory. In this regard, administration of scopolamine induces performance deficits in the radial maze (Burešová & Bureš, 1982). Peripheral administration of scopolamine can constantly interfere with short-term memory in object recognition and spatial alternation tasks (Sambeth, Riedel, Smits, & Blokland, 2007). Also scopolamine impairs memory acquisition and retention in Morris water and passive avoidance (Cozzolino et al., 1994).

It is postulated that blockade of muscarinic receptors is the main mechanism of scopolamine-induced memory impairment, but several studies argued that stimulation or blockade of other neurotransmitter systems such as glutamatergic (Gutierres et al., 2014; Mahmoodi, Ahmadi, Oryan, & Zarrindast, 2010), adrenergic (Azami et al., 2010), dopaminergic (Aalto, 2005; Dash, Moore, Kobori, & Runyan, 2007), serotonergic (Barnes & Sharp, 1999; Da Silva Costa-Aze, Quiedeville, Boulouard, & Dauphin, 2012; Dash, et al., 2007), and histaminergic systems (Chen & Kamei, 2000) could ameliorate scopolamine-induced memory impairment suggesting that these neurotransmitters may be involved in scopolamine-induced memory impairment. Also scopolamine induces oxidative stress (Jang et al., 2013), apoptosis (Jahanshahi, Nickmahzar, & Babakordi, 2013), and inflammatory responses (Ahmad, Ramasamy, Jaafar, Majeed, & Mani, 2014) in the brain tissue. So the exact mechanism of scopolamine is still unknown.

Several studies have reported that oxidative stress is one of the mechanisms involved in scopolamine-induced amnesia. Scopolamine decreases the activity of super-oxide dismutase, catalase, glutathione s-transferase, and glutathione peroxidase (Uma & Maheswari, 2014), also it increase the level of malondialdehyde marker of lipid peroxidation (Abd-El-Fattah, Abdelakader, & Zaki, 2014). Several antioxidant compounds could ameliorate scopolamine-induced memory impairment by attenuating oxidative stress markers (Harrison, Hosseini, Dawes, Weaver, & May, 2009; Hritcu, Stefan, Brandsch, & Mihasan, 2015) indicating crucial role of oxidative stress in scopolamine-induced amnesia.

Numerous studies have used scopolamine to induce memory impairment in experimental animals but the doses of scopolamine administration were different in these studies. Some used single dose and some repeated doses of scopolamine. Thus, it is not clear weather intensity and duration of scopolamine-induced amnesia differ following single or repeated doses. Because of the complex action of scopolamine it seems that mechanisms of scopolamine-induced amnesia are different following administration of single dose or repeated doses. As scopolamine is used as the reference standard drug for inducing memory impairment in experimental animals also the animal model of scopolamine is used for preclinical study of new substances in treatment of dementia, better understanding of scopolamine-induced memory impairment helps researchers better interpret the results. The present study aims to compare memory impairment and oxidative stress after administration of single or repeated doses of scopolamine.

## 2. Methods

### 2.1. Animals

Male Wistar rats were maintained in constant temperature (25°C) and 12:12 h light/dark cycle and fed with a standard laboratory diet and water. All animal experiments were approved by Ethics Committee of the Tarbiat Modares University.

### 2.2. Study materials

Scopolamine hydrobromide, Acetylthiocholin (ATCh), 5,5-dithiobis (2-nitrobenzioc) acid (DTNB), Thiobarbituric Acid (TBA), malondialdehyde bis (dimethyl acetal) were purchased from Sigma (USA).

### 2.3. Experimental design

The experimental design included two experiments: Experiment 1; and Experiment 2. In experiment 1, the effect of single dose administration of scopolamine on memory retrieval was assessed. The animals were trained in passive avoidance apparatus. About 24 hours after training, the animals received saline or different doses of scopolamine intraperitoneally (0.5, 1, and 3 mg/kg) and their memory retrieval was assessed 30 minutes and 7 days after the injection.

In experiment 2, the effect of administration of scopolamine in repeated doses on memory retrieval was assessed. The animals were trained in passive avoidance apparatus. About 24 hours after training, the animals received saline or different doses of scopolamine intraperitoneally (0.5, 1, or 3 mg/kg) for seven consecutive days and then their memory retrieval was assessed 30 minutes and 7 days after the last injection.

### 2.4. Passive avoidance test

The step-through inhibitory avoidance apparatus consisted of two compartments of the same size (20×20×30 cm) separated by a guillotine door (7×9 cm). The walls and floor of one compartment are made of white opaque resin which forms light compartment. The walls of the other compartment are black and forms dark compartment. The floor of the dark compartment is made of stainless steel bars (3 mm in diameter and 1 cm apart). Intermittent electric shocks (50 Hz for 3 s, and with 1 mA intensity) were passed on to the grid floor of the dark compartment by an insulated stimulator.

All rats were placed in the experimental room and allowed to habituate for at least 30 min before the experiments. Then each rat was kindly located in the light compartment of the apparatus. The guillotine door was opened after 5 s and the animal was allowed to enter the dark compartment. The time taken for the animal to enter the dark compartment with all four-paws (Retention time) is recorded. All rats stayed more than 120 s in light chamber and did not enter the dark compartment were excluded from the experiments. Once the rat entered with all four-paws to the dark part, the guillotine door was closed and an electrical foot shock (50 Hz, 1 mA for 3 s) was delivered through the stainless steel bars. The rat was removed from dark compartment after foot shock and trained again. The training was terminated after the rat remained in the light compartment for 120 s. The number of trials (entries into the dark chamber) was recorded. All the rats were trained with a maximum of 3 trials.

A retrieval test was carried out to determine the long-term memory formation 24 h and 7 days after the training. Each animal was placed in the light compartment for 20 s, then the door was opened and the step-through latency for entering the dark compartment (retention time) was measured. The cut-off time of 300 s was applied for those animals which still remained in the light compartment. During these sessions, no electric shock was applied.

### 2.5. Open field

The open field apparatus was made of wooden arena, 40×40 cm, enclosed by a wall of 25 cm height. The lines were drawn on the floor with a marker which divided the arena into nine equal areas (with 3×3 divisions). After administration of the highest dose of scopolamine (3 mg/kg), for seven consecutive days, each rat was placed in the center of platform. Activity was recorded for 15 minutes and speed parameter was measured with video tracking software (Ethovision XT, Nuldos, Netherlands).

### 2.6. Preparation of brain tissue samples for biochemical analysis

After training, the animals were killed by decapitation under ether anesthesia, and their brain tissues were removed and washed with ice cold 0.1 M phosphate buffer saline (pH=7.4) then the their hippocampuses were dissected out and homogenized in ice cold PBS with 1% Triton X. The homogenates were then centrifuged at 3000×g for 10 min at 4°C, and the supernatant was used.

### 2.7. Measurement of brain AChE activity

Brain acetylcholinesterase activity was measured according to Ellman method (Ellman, Courtney, Andres, & Feather-Stone, 1961). To prepare Ellman reagent, 0.1 M phosphate buffer (pH=8.0), acetylthiocholin iodide (ATCh, 75 mM) as a substrate, and 5, 5-dithiobis (2-nitrobenzioc) acid (DTNB,10 mM) were mixed in a ratio of 150:2:5. Then, 140 μL of Ellman reagent was transferred to the each well of 96 well plate. About 10 μL of brain homogenate as an enzyme source was added to Ellman reagent in the plate and the absorbance was immediately monitored during 6 min at 405 nm using a microplate reader (BIO TEK). The activity was calculated based on extinction coefficient of Thionitrobenzoation (TNB). Protein concentration in the brain homogenate was measured using the Bradford method (Bradford, 1976).

### 2.8. Measurement of lipid peroxidation

Malondialdehyde (MDA) was measured as an index of lipid peroxidation level in hippocampal tissue. The Thiobarbituric Acid Reaction (TBAR) colorimetric assay was used for the measurement of MDA level (Soodi et al., 2015). TBA reagent consisted of 3.75% TCA and 0.0925% TBA. Two volumes of TBA reagent and one volume of brain homogenate were mixed in a microtube and the mixture was incubated at 90°C for 60 min. After incubation the microtube was placed on ice to cooling. After cooling, the microtube was centrifuged at 1000 × g for 10 min and optical density of supernatant was measured in 540 nm in plate reader. MDA standard curve was established with using the stable MDA precursor, malondialdehyde bis (dimethyl acetal).

### 2.9. Statistical analysis

The results were expressed as mean±SEM. The statistical differences were analyzed by 2-way analysis of variances (ANOVA) followed by Bonferroni multiple comparisons test. The correlation between study data was analyzed by the Pearson test. The probability level less than 0.05 were considered as significant.

## 3. Results

### 3.1. Comparison of the single and repeated doses administration of scopolamine effects on memory retrieval

**Figure 1.**
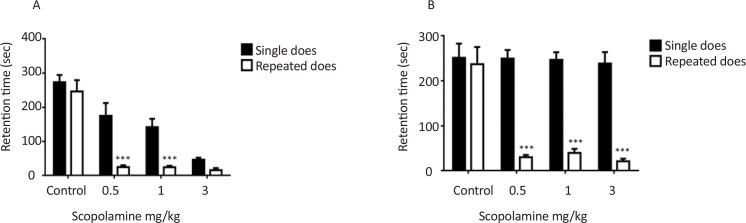
The effects of single and repeated doses administration of scopolamine on memory retrieval Rats intraperitoneally received different doses of scopolamine 24 h after training as a single dose or for seven consecutive days and retrieval test was performed 30 min after injection (A) or seven days after injection (B).

In this study, the effect of single and repeated dose administration of scopolamine on retrieval of inhibitory avoidance memory was investigated. Twenty-four hours after training, scopolamine (0.5, 1, and 3 mg/kg) was injected intraperitoneally and memory retrieval was assessed 30 minutes after injection. Figure 1A shows the results of the memory retrieval test. Significant dose effect was observed in treated groups, F_(3, 35)_=40.09, P<0.001. Single dose scopolamine dose-dependently caused memory loss, also memory impairment was observed in repeated doses treated group but this effect was not dose dependent and significant differences were not observed between different doses of scopolamine. However, significant differences were observed between effect of single dose and repeated doses of scopolamine on the memory retrieval test and repeated doses of scopolamine impair memory more potent than single dose, F_(1, 35)_=26.68, P<0.001.

Figure 1B shows the effects of scopolamine single dose and repeated doses on memory retrieval test seven days after injection. A significant effect was observed in repeated doses treated groups F_(3, 36)_=11.23, P<0.001. In single dose treated group there is no significant differences between control and treated groups and memory retrieval was not affected by single dose administration of scopolamine after seven days. In repeated doses treated groups, memory retrieval significantly was affected by scopolamine treatment and retention time significantly decreased in repeated doses treated groups compared to the single dose treated groups F_(1, 36)_=105.6, P<0.001.

### 3.2. Effect of scopolamine on locomotor activity

**Figure 2.**
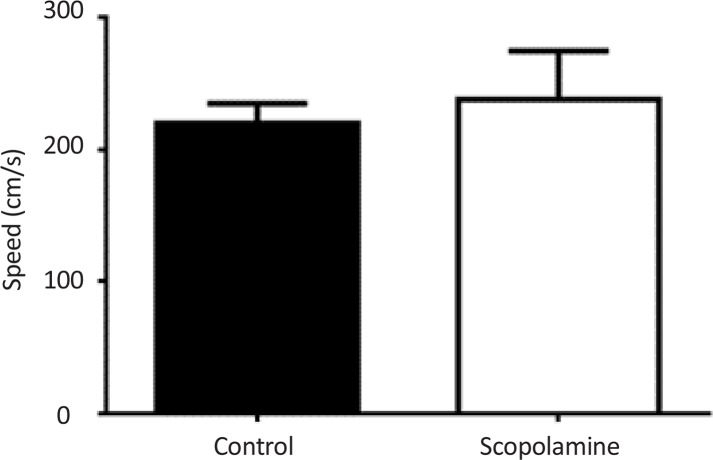
Locomotor activity after scopolamine treatment Scopolamine (3mg/kg) was administrated for seven consecutive days and locomotor activity measured by open field apparatus.

For evaluating the effect of scopolamine on locomotor activity, open field test was performed on animals which received the high dose of scopolamine (3 mg/kg) for 7 days. Results indicated that locomotor activity was not significantly different between scopolamine-treated and saline-treated groups (Figure 2).

### 3.3. Comparison of single and repeated doses administration of scopolamine on AChE activity

**Figure 3.**
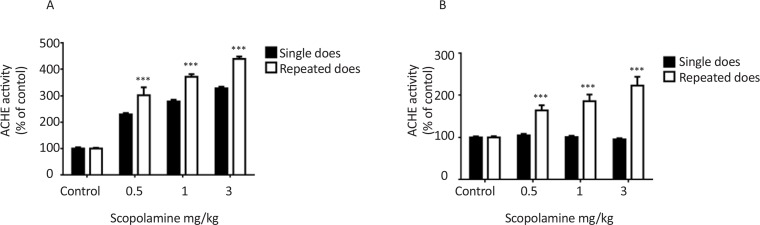
The effects of single and repeated doses administration of scopolamine on hippocampus AChE activity Rats intraperitoneally received different doses of scopolamine 24 h after training as a single dose or for seven consecutive days and retrieval test was performed 30 min after injection (A) or seven days after injection (B). *** P<0.001 represents significant differences from single dose.

Results of AChE activity in the hippocampus are shown in Figure 3. Both single dose and repeated doses administration of scopolamine significantly increased AChE activity compared to control group 30 min after injections F_(3,36)_=165.9, P<0.001. Statistical analysis indicated that AChE activity more increased in repeated doses groups than single dose groups F_(1, 36)_=70.17, P<0.001 (Figures 3A). Seven days after injection, increase in AChE activity was observed in repeated doses treated groups but not in the single dose treated groups F_(1, 36)_=66.95, P<0.001 (Figures 3B).

### 3.4. Comparison of single and repeated doses administration of scopolamine effects on lipid peroxidation

**Figure 4.**
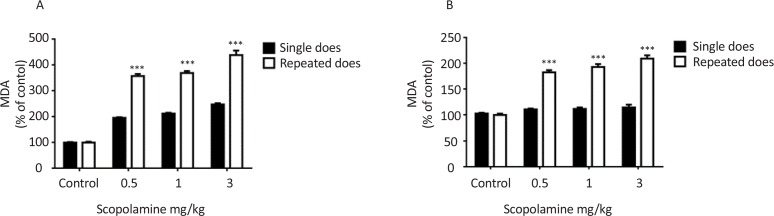
The effects of single and repeated doses administration of scopolamine on hippocampus lipid peroxidation Rats intraperitoneally received different doses of scopolamine 24 h after training as a single dose or for seven consecutive days and retrieval test was performed 30 min after injection (A) or seven days after injection (B). *** P<0.001 represents significant differences from single dose.

The Thiobarbituric Acid Reactive Substances (TBARS) are widely considered as the most common assay to measure the lipid peroxidation. Scopolamine dose-dependently increased the level of MDA in both single and repeated doses treated groups 30 min after injection F_(3, 36)_=76.25, P<0.001 but the level of MDA was more in repeated doses treated groups than single dose treated groups F_(1, 36)_=116.8, P<0.001 (Figure 4A). In single dose treated groups there is no difference in MDA level between treated and control groups seven days after injection but in repeated doses treated groups significant increase in the level of MDA was observed seven days after last injection F_(1, 36)_=117.5, P<0.001 (Figure 4B).

### 3.5. Correlation between biochemical parameters and behavioral results

**Figure 5.**
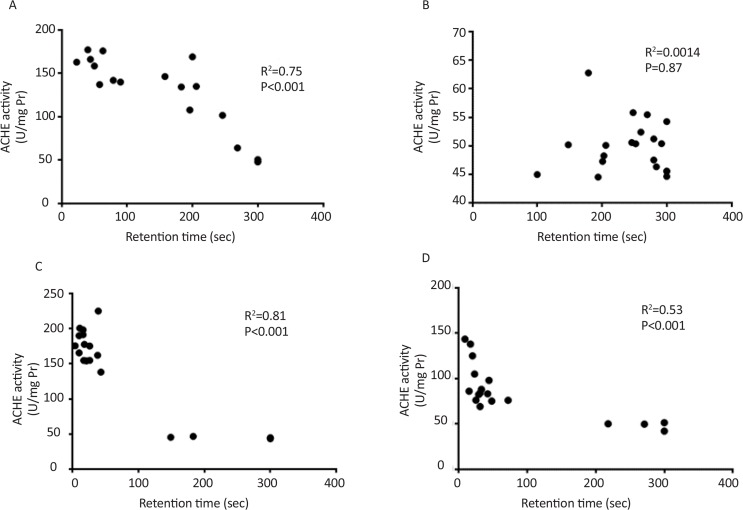
Correlation between AChE activity and behavioral test A: Single dose scopolamine administered and retrieval test was performed after 30 min; B: Single dose scopolamine administered and retrieval test was performed after 7 days; C: Scopolamine administered for 7 consecutive days and retrieval test was performed after 30 min; D: Scopolamine administered for 7 consecutive days and retrieval test was performed after 7 days.

**Figure 6.**
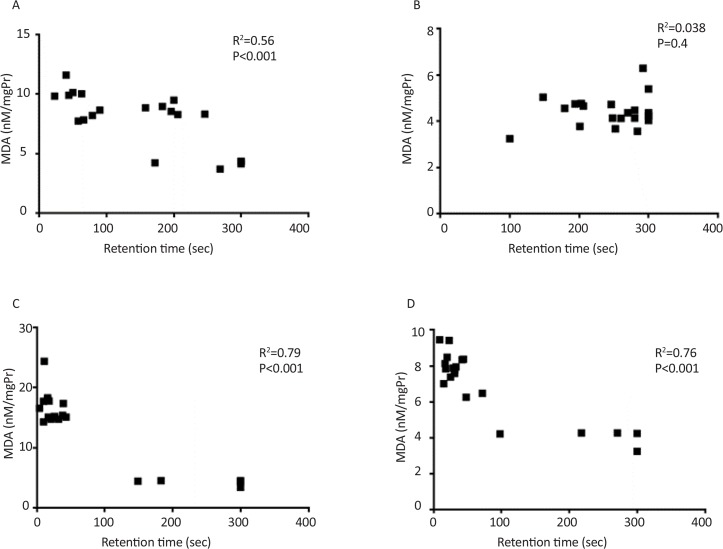
Correlation between lipid peroxidation and behavioral test A: Single dose scopolamine administered and retrieval test was performed after 30 min; B: Single dose scopolamine administered and retrieval test was performed after 7 day; C: Scopolamine administered for 7 consecutive days and retrieval test was performed after 30 min; D: Scopolamine administered for 7 consecutive days and retrieval test was performed after 7 days.

**Figure 7.**
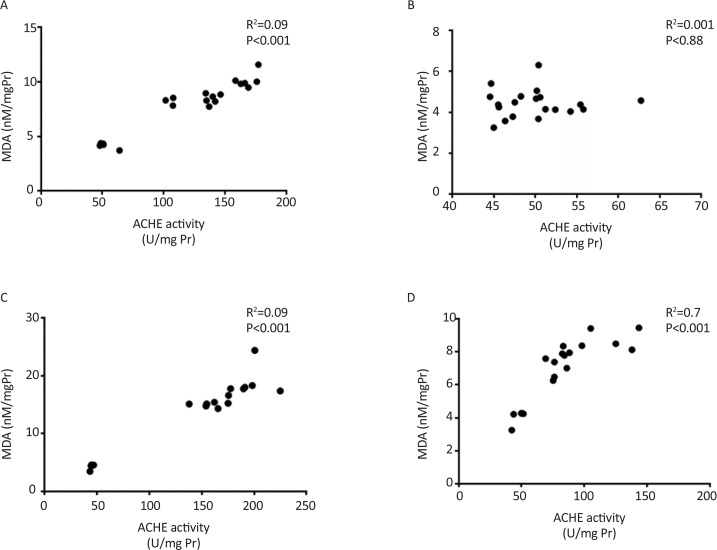
Correlation between AChE activity and lipid peroxidation A: Single dose scopolamine administered and retrieval test was performed after 30 min; B: Single dose scopolamine administered and retrieval test was performed after 7 days; C: Scopolamine administered for 7 consecutive days and retrieval test was performed after 30 min; D: Scopolamine administered for 7 consecutive days and retrieval test was performed after 7 days.

Significant correlation between retention time and AChE activity and also between retention time and lipid peroxidation were observed in all groups except single dose treated group tested seven days after injection (Figures 5 and 6). Also significant correlation was observed between AChE activity and lipid peroxidation (Figure 7).

## 4. Discussion

In the present study, effects of single dose and repeated doses administration of scopolamine on memory retrieval and brain biochemical parameters were compared. Scopolamine is widely used for induction of memory impairment in experimental animals and scopolamine-induced amnesia is used as a model for studying therapeutic agents proposed to use in AD therapy. To this end, most studies have used single administration of scopolamine which induces reversible memory impairment and some studies have used multiple administration of scopolamine, especially studies which applied Morris water maze for assessing memory performance. But there is no report about the differences in the memory impairment, its stability and also biochemical changes in the hippocampus following repeated doses and single dose scopolamine administration which was the main purpose of this study. Results of the behavioral tests indicated that single dose administration of scopolamine dose-dependently impaired memory retrieval 30 min after injection but did not affect memory retrieval seven days after injection. These results suggest that single dose scopolamine induces reversible amnesia. Also results indicate that repeated doses administration of scopolamine produce memory impairment more potent than single dose and this effect is stable even seven days after last injection. Based on these results, we can conclude that different mechanisms may be involved in memory impairment after single dose and repeated doses administration of scopolamine.

Blockade of muscarinic receptor is the main mechanism for the scopolamine-induced memory impairment; however, the involvement of other receptors has been reported, too. Chronic administration of scopolamine increases expression of M1 muscarinic and α7 nicotinic acetylcholine receptors and NR1 subunit of glutamate receptors in the hippocampus (Falsafi, Deli, Höger, Pollak, & Lubec, 2012). These receptors are co-localized in the glutamatergic synapse in the hippocampus and have crucial roles in memory formation (Marino, Rouse, Levey, Potter, & Conn, 1998). Then it is suggested that memory impairment after repeated doses administration of scopolamine is not only due to blockade of muscarinic receptor alone and changes in acetylcholine and glutamate receptors subunit expression pattern are involved in prolong memory impairment by repeated doses scopolamine administration.

Several studies reported that scopolamine-induced memory impairment is associated with increased oxidative stress within the brain (Fan et al., 2005). In the present study, significant increase in the brain level of malondialdehyde, which is the measure of lipid peroxidation, was observed 30 min after scopolamine treatment. The same result was achieved in repeated dose administration but lipid peroxidation was much more than single injection group and also lipid peroxidation was continued for 7 days in repeated dose group but significant lipid peroxidation was not observed in single dose groups 7 days after injection. These results indicate that single dose scopolamine produces reversible lipid peroxidation but repeated doses induce stable lipid peroxidation which persists at least one week. In our experiments, lipid peroxidation was well correlated with behavior deficit. Several studies have suggested that increased inflammation and oxidative stress is associated with cognitive deficits. Inflammation plays an important role in neurodegenerative disorders (Mhatre, Floyd, & Hensley, 2004).

Increased level of pro-inflammatory cytokines including TNF-α, Interleukin (IL)-1, and IL-6 are shown in the brains of patients with dementia (Akiyama et al., 2000; Fillit et al., 1991; Hauss-Wegrzyniak, Lynch, Vraniak, & Wenk, 2002). It has been reported that chronic administration of scopolamine for 14 days increased the expression of pro-inflammatory factors such as IL-1β and TNFα and inflammation is a proposed mechanism for scopolamine-induced memory impairment (Ahmad et al., 2014). It is suggested that blockage of muscarinic receptor by scopolamine might increase the expression of TNF-α in the hippocampus (Jang et al., 2013). Inflammation is associated with generation of oxygen free radicals and oxidative stress. Then it is proposed that repeated doses administration of scopolamine induces chronic inflammation and oxidative stress which resulted in prolong memory impairment.

Several studies reported that AChE activity increases after scopolamine injection (Soodi, Naghdi, Hajimehdipoor, Choopani, & Sahraei, 2014). Increased level of AChE metabolizes more acetylcholine and reduces its level in the synapses then weakens cholinergic neurotransmission which resulted in memory impairment. Studies indicate that AChE inhibitors can reverse scopolamine-induced memory impairment (Chaudhaery et al., 2010). The mechanism of AChE activity increase by scopolamine is not completely known yet. Other than the catalytic activity of acetylcholinesterase, the noncatalytic activities were assumed for acetylcholinesterase protein. Some evidence suggests that expression of this protein was induced in stress condition such as oxidative stress (Härtl, Gleinich, & Zimmermann, 2011). Then it is postulated that scopolamine-induced oxidative stress is a possible mechanism of AChE activity increase and subsequent memory impairment. In our study, AChE activity increase was well correlated with memory deficit. Also similar to lipid peroxidation marker, increased level of AChE activity was more predominant in repeated dose group and persist at least seven days after last injection and increase in AChE activity well correlated with lipid peroxidation.

Our study indicated that repeated doses of scopolamine administration impair memory function worse and more prolong than single dose and chronic oxidative stress may be the possible mechanism. Because chronic inflammation and oxidative stress have been observed in the brain of AD patient it seems that administration of repeated doses of scopolamine better simulate the AD condition and it is recommended that chronic administration of scopolamine is used for preclinical testing of new substances designed to treat AD.
